# Optically Non-Contact Cross-Country Skiing Action Recognition Based on Key-Point Collaborative Estimation and Motion Feature Extraction

**DOI:** 10.3390/s23073639

**Published:** 2023-03-31

**Authors:** Jiashuo Qi, Dongguang Li, Jian He, Yu Wang

**Affiliations:** 1 Science and Technology on Electromechanical Dynamic Control Laboratory, Beijing Institute of Technology, Beijing 100081, China; 2 School of Mechatronic and Electrical Engineering, North University of China, Taiyuan 030051, China

**Keywords:** cross-country skiing, deep learning, movement monitoring, motion recognition, intelligent sports

## Abstract

Technical motion recognition in cross-country skiing can effectively help athletes to improve their skiing movements and optimize their skiing strategies. The non-contact acquisition method of the visual sensor has a bright future in ski training. The changing posture of the athletes, the environment of the ski resort, and the limited field of view have posed great challenges for motion recognition. To improve the applicability of monocular optical sensor-based motion recognition in skiing, we propose a monocular posture detection method based on cooperative detection and feature extraction. Our method uses four feature layers of different sizes to simultaneously detect human posture and key points and takes the position deviation loss and rotation compensation loss of key points as the loss function to implement the three-dimensional estimation of key points. Then, according to the typical characteristics of cross-country skiing movement stages and major sub-movements, the key points are divided and the features are extracted to implement the ski movement recognition. The experimental results show that our method is 90% accurate for cross-country skiing movements, which is equivalent to the recognition method based on wearable sensors. Therefore, our algorithm has application value in the scientific training of cross-country skiing.

## 1. Introduction

Due to the different energy costs of sub-movements in different terrains, skiers switch between sub-technical movements [1], which is one of the most important factors in determining competition results. Therefore, the accurate identification of cross-country skiing technical movements can effectively help athletes to optimize their gliding strategies and improve their technical movements, thus improving their performance. There are three main methods for cross-country skiing technical movement recognition [2]: one is based on improving athletes’ equipment [3,4], but cross-country skiing has high demands on equipment and professional athletes need a long time to adapt to the modified equipment, which limits the promotion and application of this method. Another is based on wearable micro-sensors [3,5,6], such as Global Navigation Satellite System (GNSS) data and seven Inertial Measurement Units (IMUs), which are used to analyze different cycle characteristics and sub-technology types during skiing. The excessive number of sensors used in this method makes it too complex to wear. At the same time, it is uncomfortable for the athlete and cannot be worn during official competitions, which limits its application. Lastly, there is the method of movement recognition based on computer vision [7]. Its main problem is that it has certain limitations in the field and requires early calibration, professional training, and time-consuming preparation. At the same time, due to the limitation of the viewing angle and the influence of the stadium environment, video recording usually does not produce satisfactory results. Therefore, in the field of cross-country skiing training and analysis, there is a need for a simple and non-contact method to capture, analyze, and identify athletes’ movements in order to improve the scientific training standards of cross-country skiing.

Smart sensors and computer vision can use deep learning technology to implement posture estimation and motion recognition [7,8,9,10,11], such as smart watches, wristbands, wearable sensors, and high-definition cameras. Inspired by the application of artificial intelligence technology in image recognition and data analysis, the introduction of machine learning into the motion state analysis of moving skiers will help to obtain more accurate and realistic ski technical action features and the motion performance of mobile personnel [12]. In the process of understanding the action cycle and motion attitude of moving skiers, image sensors are first used to collect the information of the mobile personnel, and then neural networks are used to extract the feature information. According to the extracted feature information [13], appropriate analysis or typical value feature information is selected as input information in the action understanding stage. Finally, machine learning algorithms are used to automatically identify and accurately classify the typical features or stages and output the results. In this way, the acquisition of the pole movement of the moving skiers and the detection of the ski movement can be implemented.

The challenges faced by monocular vision in human pose estimation in cross-country skiing include three main aspects: the athletes’ flexible posture, the snowfield environment, and the constraints of camera vision. Firstly, the flexible athlete posture means that there is a more complex internal relationship between key points, and the degree of freedom of body movement is higher, which poses a greater challenge for estimation. Second, the complex track environment and outdoor location may make it difficult to extract foreground information, which greatly increases the detection difficulty [14]. Thirdly, the image recognition results based on the heatmap have positioning errors and estimation errors, making segmentation and motion recognition difficult.

At the same time, the method of using images to implement motion recognition requires manual labels and training in advance [12]. These hand-crafted datasets can often only be applied to specific problems. There are problems in applying them to real scenes. In addition, the traditional manual architecture for video motion analysis can only extract low-level features, and it is difficult to extract and use high-level semantic information.

Given the difficulty of human posture estimation and motion recognition, OpenPose proposes Part Affinity Fields (PAFs) to improve the accuracy of posture estimation [9]. Marian Bittner proposed a new end-to-end method for estimating human kinematics directly from video [15]. Nguyen combined best practices at each stage of the estimation process to achieve an accurate estimation of the human body [16]. Hu proposed a coordination feature and strengthened the attention of the model, improving the accuracy of action recognition [17]. Some of these methods are based on indoor datasets, while others are based on routine human actions. Cross-country skiing is mainly practiced outdoors in a wide area, which results in some limitations of some methods, such as limited field of view and sunlight interference [18]. In addition, skiers move quickly and in complex ways, which are different from daily movements. Few existing algorithms have been studied for a situation where the above challenges occur simultaneously.

Considering the problems of applying pose estimation in real scenes and the current situation wherein it is difficult to extract and use advanced semantic information in motion video analysis, it is difficult to apply an image-based pose analysis method to support cross-country skiing training. In this paper, a monocular pose recognition method for cross-country skiing is proposed. This method simultaneously detects the position and key points of the human body, estimates the depth information of the key points, and then uses the time domain features and frequency domain features to expand the feature dimension and fuses the recognition results of the support vector machine (SVM) and K-nearest neighbors algorithm (KNN) to improve the accuracy of motion recognition.

The main innovations of this article are as follows:

1. This paper proposes a cross-country skiing technical action recognition method based on an image sensor, deep learning, and sub-action interval classification, which implements the effective classification of sub-actions and can provide data support for athletes to optimize sliding strategy and improve technical actions.
2. By simultaneously detecting the positions of key points and the positions of human targets, a three-dimensional human posture estimation method without a heatmap is implemented. When identifying key points, four feature layers of different output grid scales are used to simultaneously detect human posture and key points. Constraining key points using human body detection regions, the key points are identified only in the area around the target candidate, avoiding interference from the external environment. At the same time, it reduces the error caused by low resolution and close key points in the heatmap method. Compared with heatmap-based recognition methods, it is more suitable for detecting two-dimensional key points on the human body in outdoor environments. By using accurate two-dimensional key-point information as input, three-dimensional position estimation can obtain precision information about the depth of key points and reduce recognition errors by position offset loss and rotation compensation loss.
3. According to the characteristics of cross-country skiing, the cross-country skiing action characteristics are divided. Through the classification and feature extraction of key points, non-contact automatic cross-country skiing technical action recognition can be implemented.

## 2. Related Works

In this section, we will review, in detail, the progress of ski motion analysis technology and the development of human behavior recognition methods based on neural networks.

The motion recognition of cross-country skiing technology mainly depends on sensors and image processing.

The sensor-based ski posture recognition methods mainly collect motion information through wearable and modified ski equipment. Cenedese et al. achieved ski sub-action recognition based on wrist position IMUs and a relevance vector machine (RVM) algorithm [19]. Rindal et al. used arm and chest data from IMUs and neural network classification to achieve ski motion recognition with an average accuracy of 94% [20]. Marsland et al. used sensors integrated with GNSS and IMUs to integrate velocity, angular velocity, and motion frequency features to achieve sub-action recognition [21]. Johannsson et al. added a force sensor and IMUs to the ski pole to achieve ski sub-action recognition with 78% accuracy [22]. Meyer et al. designed a snowboard with 10 sensors to measure ski force and torque [23]. According to the above example, the sensor-based ski motion detection method can achieve motion detection. However, there are some problems, such as complex wearing, large volume, and the weight impact of sensors that cause training interference and sports injury. At the same time, if there is an error in the fixed position of the sensor, the recognition accuracy will be greatly reduced.

Fasel et al. used the motion capture system based on 10 infrared thermal cameras on the indoor ski carpet to obtain the relative position of the marked points on the athletes and the kinematic parameters of the center of mass [5]. Kruger et al. used three synchronous cameras (50 Hz) to capture the athlete’s posture during the 1.5 m turn [24]. The EPFL has created an alpine skiing dataset to solve the problem of data scarcity [25]. Gastaldi et al. carried out the unmarked motion analysis of skiing using the motion analysis method based on feature detection and point tracking [26]. Due to the nature of the game and the stadium setting, only one camera can be used and only 2D kinematics analysis can be performed. The Norwegian Institute of Sports and the University of Ljubljana in Slovenia developed a ski posture analysis system that analyses the key technical movements of local positions through training videos to improve the competitive level of athletes [27]. The University of Zurich implemented the three-dimensional reconstruction of skiers’ postures using beam adjustment [28]. Keizo used motion capture technology to study the complexity of ski jumping and reduce the effects of resistance [29]. Although these motion capture systems are very accurate, they are limited by the shooting area and the environment. The camera needs a line of sight to see the target, which limits the movement and the movement that can be studied. At the same time, some motion detection systems are effective for indoor experiments, but the use of such systems for outdoor sports has some limitations. For example, in outdoor skiing, such systems can be affected by the reflection of sunlight off the snow. In addition, in cross-country skiing and other snow events where several people are sliding for a long time, it is not easy to identify the required information from the video, the post-processing workload is high, and real-time feedback is rarely achieved. Considering the hardware cost and technical difficulty of the optical acquisition system in the snow field, the target recognition and pose estimation method based on deep learning can provide a new solution for vision-based ice and snow motion monitoring.

Motion recognition based on abstract 3D skeletons has become an active topic in the field of computer vision due to its potential advantages. Wang [30] proposed a Joint Trajectory Map (JTM), which represents the configuration and dynamics of space joint trajectory through color-coded texture images. Yanshan and Rongjie [31] put forward the representation of shape and motion in geometric algebra. According to the representation, the importance of joints and bones has been fully utilized, which makes full use of the information provided by bone sequences. Therefore, only the adjacent joints in the convolution kernel are considered to learn the co-occurrence feature; that is, for each key point, some potentially relevant key points will be ignored. Tran et al. proposed a modern deep architecture of C3D (revolutionary 3D), which can learn from large-scale data sets and integrate the outputs of different structures for prediction [32]. Wei proposed an end-to-end recursive neural network based on a skeleton [33]. According to the physical structure of the human body, the human skeleton is divided into five parts and sent to five subnetworks, which can well model the contextual information of a time series [34]. Wang et al. made the key points of different degrees of freedom (DoF) monitor each other, resulting in physical constraints and reasonable attitude estimation results [35]. Ma et al. proposed ContextPose, based on an attention mechanism, which can more accurately estimate 3D poses by implementing soft limb length constraints in the depth network [36]. With the development of deep learning, compared with traditional algorithms, deep learning algorithms can not only automatically extract features but also perform well in many cases. However, it is still difficult to effectively recognize outdoor scenes and complex human movements. In the outdoor environment, the angle of vision is limited and there are obstacles. These difficulties cause the key points of the human body to be easily blocked or the human body to adopt a side posture in the field of view. The lack of too many key points will affect the posture estimation results. At the same time, the posture of the human body is flexible in movement, and each person’s figure is different, so it is difficult to describe different postures. Some of the above literature methods improve the estimation effect by improving the key-point recognition accuracy, and some improve the estimation accuracy by adding constraints. In all the estimation difficulties due to the flexibility of outdoor environments such as that of cross-country skiing, it is necessary to improve the key-point recognition accuracy, angle of view, and other constraints to better achieve the attitude estimation.

In general, the optical motion capture system has the advantage of non-contact motion parameter acquisition. However, it can only capture a small sliding range or requires a large hardware investment. Therefore, optical motion detection still has some shortcomings in outdoor applications. In human posture estimation and motion recognition, it is still difficult to effectively recognize outdoor scenes and complex human movements. Therefore, outdoor ski motion estimation based on a monocular camera and a neural network has research value and significance in ski science training.

## 3. Method

In this section, we analyze the actual needs of monocular cameras used for cross-country skiing motion recognition and describe the research methods proposed, including the algorithm framework, the target detection method, the three-dimensional pose estimation algorithm by two-dimensional joint detection, and the sub-motion recognition method.

### 3.1. Requirements and Analysis of Sub-Action

**Figure 1:**
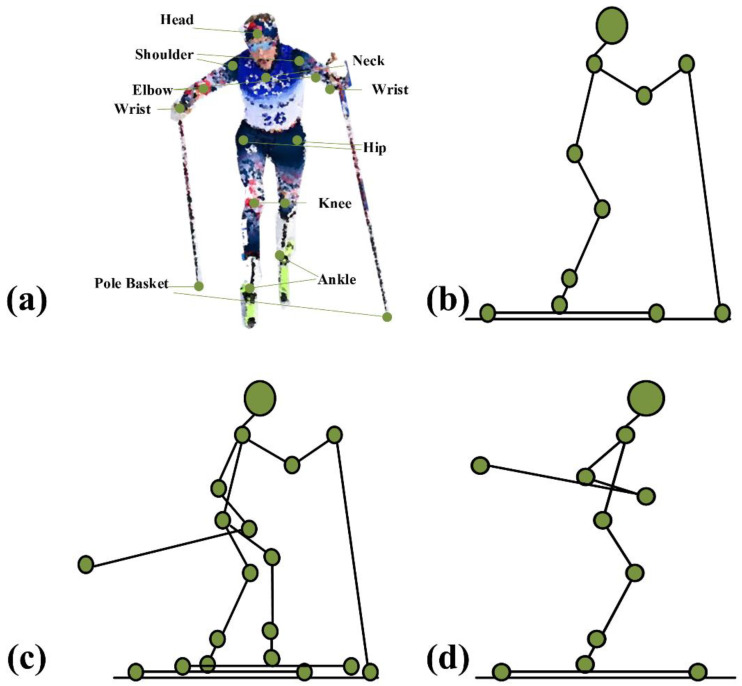
The key-point position and skiing sub-actions. (**a**) shows the key points of our work. (**b**) shows the DP action. (**c**) shows DS action. (**d**) describes the DT action.

The first step is to analyze the real needs of scientific training in cross-country skiing. Cross-country skiing is essentially divided into three typical sub-actions. These sub-actions mainly include double poling (DP), diagonal striding (DS), and downhill techniques (DT) [37]. The DP sub-action means the athletes slide the ski poles at the same time and the legs on both sides also move synchronously, as shown in Figure 1b. During the DS action, the athletes’ poles are poled alternately, while the two legs move forward alternately, as shown in Figure 1c. In DT, in Figure 1d, the ski poles are clamped between the athlete’s arm and body to bend and tuck. The technical actions have a decisive impact on the competition results of skiing. The technical action selection, technical action cycle, and sliding rhythm of cross-country skiing will directly affect the performance of skiing in training and competition. The research shows that the changes in technical tactics and training strategies can significantly reduce the metabolic cost per meter of cross-country skiing [38]. By optimizing performances under different terrains based on feedback, athletes can improve competition results. Considering each method, the ski pole position and the ankle joint position are different at the same time, and the changing trend of the joint angle is different. The ankle joint position, ski pole position, and knee joint angle at the same time are used as the characteristic signal input to the algorithm. By matching the ski pole position information and the human body movement parameters, the classification of technical actions and the recognition of the movement phase are analyzed to achieve an understanding of skiers’ movements and improve the scientific training level. According to the characteristics of the different sub-actions, the following classification can be made, as shown in Table 1.

According to the results of sport biomechanical analysis of cross-country skiing, the range of motion of elbow and shoulder joints, angular velocity, and the angle between the snowball and the ground are all related to sports performance. In addition, the coordination of joint activities is also conducive to improving work efficiency and athletic performance. At the same time, the coordination between the shoulder, elbow, and trunk can cause the body to have a large inclination change, which is conducive to the active extension of the elbow joint, thus generating greater propulsion [39]. Therefore, according to the characteristics of the movement, we can build a physiological model and analyze the kinematic model to implement pose recognition. The joint angle used in this article is calculated from the spatial position between coordinate points in space. The kinematic model consists of 17 anatomical points corresponding to the right (left) temple, shoulder, elbow, wrist, hip, knee, and ankle, respectively; in addition, two additional points are added: the bottom point of the snow poles, as shown in Figure 1a.

**Figure 2:**
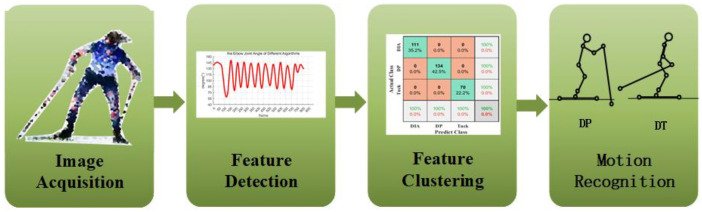
Architecture of monitoring recognition for skiing state.

**Figure 3:**
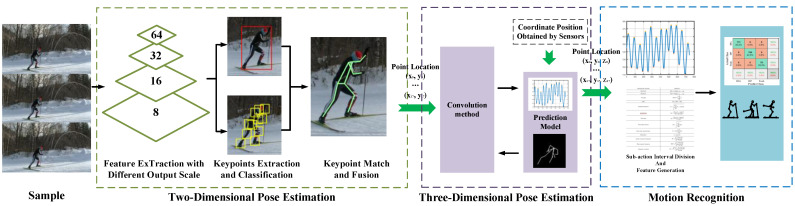
The framework of our algorithm. The algorithm consists of three modules: a two-dimensional joint estimation module, a three-dimensional pose estimation module, and a motion recognition module.

To sum up, monocular outdoor motion recognition faces three major challenges: different attitude displacement scale transformation, attitude size scale transformation, key-point noise, and recognition errors caused by missing key points. To solve these problems, we designed an action recognition algorithm based on multiple neural networks and feature generation, as shown in Figure 2. First, a human body detector was designed to find the target human body and each human candidate region by detecting key points in the image or video. A similar top-down detection method does not easily generate interference between different joint points. The depth information was obtained by calculation or prediction, and the three-dimensional key-point coordinates were obtained by using the depth information to implement the three-dimensional construction of human posture. Finally, the attitude data were divided into sub-action regions according to prior knowledge and feature generation was performed. Finally, the generated feature information was divided into sub-action regions using support vector machines, as shown in Figure 3. The whole algorithm framework is made up of three modules: a key-point region detection module based on YOLO [40], a 3D pose estimation module, and an action recognition module.

### 3.2. Two-Dimensional Pose Estimation Module

Currently, most mainstream human posture estimation methods take high-resolution images as input and use a Gaussian heatmap to estimate the position information of key points. The research shows that in the same model, the higher the resolution of the input image, the higher the recognition accuracy of the heatmap [41]. The accuracy of key-point prediction is essentially limited by the resolution of the heatmap, which leads to the accuracy problem of the commonly used heatmap method. At the same time, Feature Pyramid Networks (FPNs) are often used in the recognition process to restore the size of the input. In the outdoor environment, the distance between the camera and the target is often large, and the proportion of the target in the field of view is small. Therefore, the true resolution of the target area is low, the accuracy of the heatmap is reduced, and there is a feature shift in the feature layers of different scales, which increases the uncertainty of the key-point location. For example, if two key points of the same category are close to each other, they may be mistaken for the same key point due to overlapping heatmap signals, leading to key-point detection failure and affecting the final recognition effect [42,43]. Given the obvious shortcomings of the heatmap, this work calibrates the human body and joints based on YOLOv5, does not use a heatmap, and contains an efficient network design. It simultaneously detects the target and its key points at the same time and uses a matching algorithm to fuse the two kinds of results.

The two-dimensional pose recognition module in this article uses a CNN to extract features and then uses the full connectivity layer to regress the coordinates of key points by MSE (mean square error) loss. During pre-training, the input training set was classified into different key points of the human body, and the output has 18-dimensional object class scores, including the key-point categories and the person category. All key points are computed simultaneously and share the same feature information. For each grid scale in the image, the confidence is calculated for all categories, and the target category has the highest confidence in the location.

The output grid scales of YOLO are 8, 16, 32, and 64, selected by the hypermeters of reference [42]. Each grid uses different anchors. The smaller grid has a larger receptive field and can predict larger objects; the larger grid has a smaller receptive field and is more suitable for predicting smaller objects.

In this paper, the multi-task loss function was used to train the network, which mainly includes the loss of probability of target existence, the loss of boundary box size, and the loss of category score. The loss of each task is calculated as follows:

(1) $$Loss_{2d}=a\times Loss_{obj}+b\times Loss_{iou}+c\times Loss_{clc}$$

where category loss, confidence loss, and positioning loss are represented by $Loss_{obj}$, $Loss_{iou}$, and $Loss_{clc}$, respectively, and a, b, and c are shown as the balancing coefficients.

The category loss is calculated using binary cross entropy. In this paper, there are 18 kinds of joint points and human targets and a total of 19 kinds of targets. The formula is as follows:

(2) $$Loss_{obj}=\sum_{s}BCE\left(c_{i},c_{g}\right)$$

where $c_{i}$ and $c_{g}$ are used to represent the category of the target identified by the algorithm in this paper and the truth label of the target, respectively.

The confidence loss is used to describe whether there is a center point on this grid; that is, whether there is an object. The algorithm in this paper regards confidence as a two-class problem. The closer the predicted value is to 1, the more likely it is that there is a target at that location, and the less likely it is that there is no target. The formula is as follows:

(3) $$Loss_{cla}=\frac{\sum_{i\in AK}\sum_{j\in clc}\left(G_{ij}\ln({\hat{C}}_{ij})+\left(1-G_{ij}\right)\ln(1-{\hat{C}}_{ij})\right)}{N}$$

(4) $${\hat{C}}_{ij}=Sigmoid\left(C_{ij}\right)$$

(5) $$G_{ij}\in \left\{0,\;1\right\}$$

where N is used to describe the number of targets, AK is used to describe the total number of bounding boxes, and $G_{ij}$ is used to indicate whether there is a type j target in the bounding box i or not. ${\hat{C}}_{ij}$ is used to indicate the probability of the existence of the type j target in the boundary box i detected by our method.

The calculation of location loss is mainly to improve the accuracy of the detection method in this paper. The d is used to describe the prediction box. $d_{x}$, $d_{y}$, $d_{w}$, and $d_{h}$ are used to show the center-point coordinates and the width and height values of the prediction box, respectively. We use $g_{x}$, $g_{y}$, $g_{w}$, and $g_{h}$ to represent the true center-point coordinates and width and height, respectively. The location loss is calculated by the total number of samples and the sum of squares of the difference between the target positions estimated by the algorithm and the target positions labeled by the truth value. The formula is as follows:

(6) $$Loss_{iou}\left(d,g\right)=\frac{\sum_{i\in AK}{\left(\sigma \left(d_{x}{}^{i}\right)-g_{x}{}^{i}\right)}^{2}+{\left(\sigma \left(d_{y}{}^{i}\right)-g_{y}{}^{i}\right)}^{2}+{\left(d_{w}{}^{i}-g_{w}{}^{i}\right)}^{2}+{\left(d_{h}{}^{i}-g_{h}{}^{i}\right)}^{2}}{N}$$

For the anchor, the general height of the human body is 3–4 times the width, and the commonly used square hyperparameter anchors are obviously not suitable for the research content of the subject. Therefore, instead of using the basic hyperparameters, we modified the default values of the anchors and used the width-to-height ratio of 1:4 for the human target.

(7) $$b_{x}=\left(2\sigma \left(d_{x}\right)-0.5\right)+AK_{x}$$

(8) $$b_{y}=\left(2\sigma \left(d_{y}\right)-0.5\right)+AK_{y}$$

(9) $$b_{w}=AK_{w}\times e^{d_{w}}$$

(10) $$b_{h}=AK_{h}\times e^{d_{h}}$$

where $b_{x}$, $b_{y}$, $b_{w}$, and $b_{h}$ are used as candidate boxes predicted by the algorithm in this paper. $d_{x}$, $d_{y}$, $d_{w}$ and $d_{h}$ are used to describe the predicted regression parameters. $AK_{x}$, $AK_{y}$, $AK_{w}$ and $AK_{h}$ are used to describe the coordinates, width, and height of the anchor. σ is shown as the sigmoid function.

Non-maximum suppression is applied to the training results, and the scores of all the boxes are calculated. The candidate boxes are sorted by confidence and then the intersection over union (IOU) is calculated with the highest confidence candidate box, respectively. If it is greater than the set threshold, the box is deleted.

To determine which target the key points belong to, this paper uses a fusion method. After detecting human targets (candidate regions) and key points, if there are candidate regions, the Euclidean distance between all key points and the center of the candidate region is calculated. For each type of key point, the result of the minimum distance from the candidate region is selected as the key point of the target.

When training the two-dimensional pose estimation module, the three hyperparameters of the balancing coefficients were 0.6, 0.2, and 0.3, respectively. The hyperparameters of the human target anchors for the 8 scale were (12, 20), (26, 46), and (43, 102), respectively. The module was trained by 500 epochs. The training set used by the network consisted of three parts: the COCO dataset images containing both the person and snowboard categories, part of the COCO dataset images containing the person category, and the cross-country skiing dataset labeled in this work.

In 2D human pose estimation, the key points are projected onto the two-dimensional plane and the key-point detection results have no depth information. Three-dimensional pose estimation adds depth information based on 2D pose estimation, and the key points are expressed more accurately [44]. Its application research value in ski motion recognition is higher than 2D estimation. Therefore, after extracting accurate 2D key-point coordinate information, we designed a 3D key-point estimation strategy to estimate the depth information.

### 3.3. Three-Dimensional Pose Estimation Module

We designed a 3D key-point estimation strategy. First, in order to finally ensure that the target contains a complete person, we take the head and neck as the root feature and use each head and neck as a marker to read more complete pose information, so as to ensure the existence of the candidate targets according to the head and neck. Then, according to the completed two-dimensional key-point data, the depth information of each key point is predicted through the network, and the three-dimensional depth information of each key point is obtained. Finally, according to the predicted joint information, a complete human posture is formed from the root node to implement the three-dimensional posture estimation. If any key points are lost, the 3D position information of the missing key points in the front and back frames is used to supplement the missing information using the least squares method. The reason why the system chooses to use the least squares method is that the video frame rate is higher than 50 Hz and the position of the key-point position information in the adjacent frames does not change much, and the second reason is that the human key-point motion attitude is relatively linear. We use the head key point and neck key point as root features, and use each head and neck as a marker to read more complete pose information, so as to ensure the existence of candidate targets based on the head and neck. Then, according to the completed two-dimensional key-point data, the depth information of the key points is predicted through the network, and the three-dimensional depth information of each key point is obtained to achieve the depth information prediction. Considering the position deviation loss and rotation compensation loss of key points, the total loss function ($Loss_{total}$) used for the training module is defined as

(11) $$Loss_{3d}=Loss_{location}\left(X\right)+Loss_{rot}$$

where $Loss_{location}\left(X\right)$ represents the offset of key points, and $Loss_{rot}$ is described as a rotation error. The $Loss_{location}\left(X\right)$ is shown as follows:

(12) $$Loss_{location}\left(X\right)=\frac{1}{N}\sum_{i=1}^{N}Loss_{location}\left(X\right)$$

where X is shown as the total loss of all targets and $x_{i}$ represents the loss of each joint of each target. The loss of rotation is described by Formula (7).

(13) $$Loss_{rot}=\frac{1}{N}\sum_{i=1}^{N}{\left(R^{i}-R^{iGT}\right)}_{2}$$

(14) $$R^{i}=R_{x}^{i}\cdot R_{y}^{i}\cdot R_{z}^{i}$$

where $R^{i}$ is used to describe the rotation matrix in three directions of $R_{x}^{i},R_{z}^{i}$, and $R_{y}^{i}$. $R^{iGT}$ is used to represent the true value of the rotation matrix.

We used the network structure and method of [45] with ResNet-50 [46] as the backbone, applied Euclidean loss, and used 12 of the 14 available camera views in the MPI-INF-3DHP (MPI) dataset for training [47]. For training, we crop around the subject closest to the camera and apply rotation, scaling, and bounding box jitter enhancement. We use a size of 6 batches and the cycle learning rate ranges from 0.1 to 0.000001.

This work estimates the position of human joints in three-dimensional space using two-dimensional key-point coordinates as input. During training, the 3D pose estimation network uses the MPI-INF-3DHP (MPI) dataset for training. In application, our input is a series of two-dimensional points and our output is three-dimensional coordinates with depth information as reference [48]. It uses the AdaDelta solver, with a momentum of 0.9, a weight decay multiplier of 0.005, and a batch size of 6, and trains the model for 360k iterations with a cyclical learning rate ranging from 0.1 to 0.000001.

**Figure 4:**
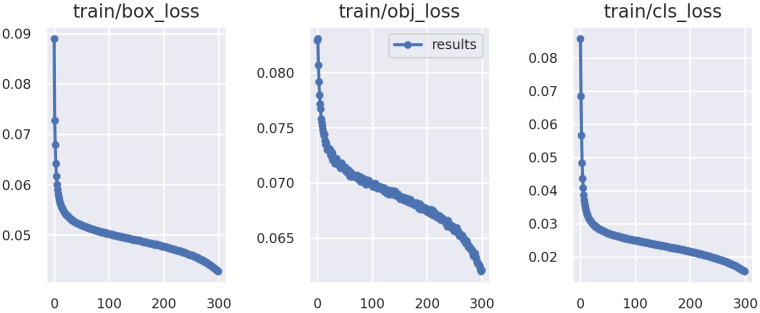
The loss curve of our method.

The loss curve is shown in Figure 4. It can be seen that our curve gradually converges smoothly, and the convergence curve reflects the performance of the algorithm.

**Figure 5:**
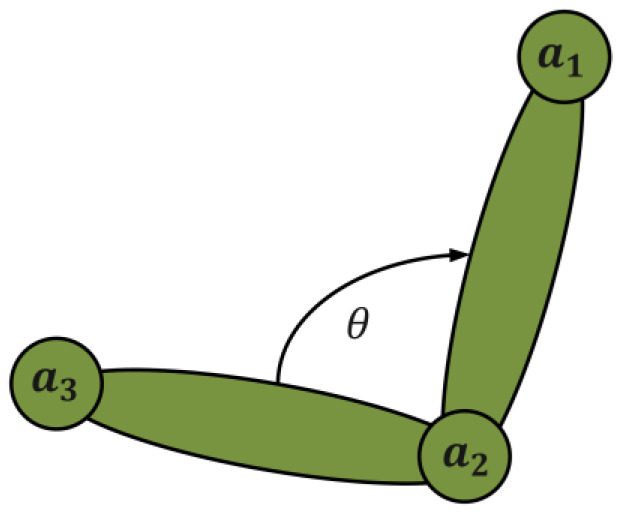
The schematic diagram of the key points.

The algorithm estimates the depth information of the key points using a pre-trained model (Figure 5). In the actual estimation, there are still some key points that cannot be predicted in occlusion and target deformation. The video frame rate is 60 Hz, and the human movement in each frame is relatively close. Therefore, through linear interpolation on the tensor product mesh of 3D discrete predicted data, the position of the key point can be obtained by using the position information of adjacent frames. The unrecognized joint point coordinates are ($x_{m},\;y_{m},\;z_{m}$), and the formula is as follows:

(15) $$x_{m}=(x_{1}-x_{0})/2$$

(16) $$y_{m}=(y_{1}-y_{0})/2$$

(17) $$z_{m}=(z_{1}-z_{0})/2$$

where $x_{1},\;y_{1}$ and $z_{1}$ represent the position information of key points in the next frame, and $x_{0},\;y_{0}$, and $z_{0}$ represent the key-point information of the previous frame of the lost frame, respectively. In this way, the missing key points can be supplemented, and the success rate of subsequent action feature recognition can be improved to a certain extent.

In the pose evaluation, each joint angle in the movement process will significantly affect the movement posture, and the movement posture will significantly affect the mobility. Therefore, in the process of maneuvering, obtaining information on the athlete’s posture joint angle is conducive to assisting the coach in improving the athlete’s mobility. The joint angle used in this article is calculated from the spatial position between coordinate points in space. After extracting the position of each joint point in 3D space, this paper calculates the angle between joints. In this process, the connection between joint points is regarded as a chain structure in which the local stiffness is not changed. Take the right elbow joint as an example, as shown in the Figure 5, where $a_{1}$, $a_{2}$, and $a_{3}$ represent the right shoulder joint, right elbow joint, and right wrist joint. The angle of the elbow joint is the included angle with the right elbow joint as the vertex θ. The calculation is publicized as follows:

(18) $$\theta =\cos^{-1}\frac{\langle \vec{a_{2}a_{1}},\vec{a_{2}a_{3}}\rangle }{\left|\vec{a_{2}a_{1}}\right|\left|\vec{a_{2}a_{3}}\right|}$$

### 3.4. Action Recognition Module

In the experiment, the predicted joint trajectory is not smooth enough, and the joint position produces jitter. It is impossible to easily identify the key frames related to the feature stage by the spatial position information of the coordinate points. To reduce the effect of this kind of jitter, this paper uses the expectation maximization (EM) algorithm for parameter estimation and the Butterworth filter to achieve accurate joint recognition and jitter suppression.

The EM algorithm consists of two steps, which are called expectation (E step) and maximization (M step). Step E calculates the posterior probability of implicit variables according to the initial value of parameters or the model parameters of the previous iteration, which is actually the expectation of implicit variables. As the current estimated value of hidden variables, it is calculated as follows:

(19) $$Q_{i}\left(z^{\left(i\right)}\right):=p\left(z^{\left(i\right)}|x^{\left(i\right)};\theta \right)$$

where $Q_{i}\left(z^{\left(i\right)}\right)$ is used to describe the posterior probability. $x^{\left(i\right)}$ and $z^{\left(i\right)}$ are used to represent input and response, respectively. The initialization distribution parameter uses *θ* express. The solution is the maximum likelihood estimate of *θ*, as follows:

(20) $$\theta :=arg\underset{\theta }{\max}\sum_{i}\sum_{z^{\left(i\right)}}Q_{i}\left(z^{\left(i\right)}\right)\log\frac{p\left(z^{\left(i\right)}|x^{\left(i\right)};\theta \right)}{Q_{i}\left(z^{\left(i\right)}\right)}$$

Through continuous iteration, the lower bound continues to increase, and the maximum likelihood estimation shows a monotonic increasing trend, eventually reaching the maximum value of the maximum likelihood estimation. This step is called the M step.

In practical applications, the ultimate goal is to achieve the detection and understanding of the key-point location and sub-action. In order to reduce the negative impact of the data jitter caused by each frame recognition result on the detection result, we also use the Butterworth filter to further reduce jitter. The characteristic of the Butterworth filter is that the frequency response curve in the passband is as flat as possible without fluctuation, which can effectively reduce the interference in the process of motion, which is more suitable for the purpose of finding poles. The formula of the Butterworth filter is as follows:

(21) $${\left|H\left(\omega \right)\right|}^{2}=\frac{1}{1+{\left(\frac{\omega }{\omega_{c}}\right)}^{2n}}$$

where H(ω) is the result of passing the Butterworth filter, and ω is the corresponding input. The order of the filter is expressed by n, and the cut-off frequency is shown as $\omega_{c}$. We have tested that the third-order Butterworth filter has a good effect. Too high an order of the Butterworth filter will result in too flat a curve and pole loss, which is not conducive to improving the detection success rate and detection accuracy. The low order of the Butterworth filter will still cause the jitter of the characteristic curve, which can easily lead to false detection. After the parameter test, the third-order Butterworth filter, the cut-off frequency is 14 Hz, the key-point curve is relatively flat, and the information storage is also rich.

After the motion path has been smoothed, the x-coordinate of the time series is clustered into segments representing the standing stage or the pole stage. It is assumed that in the standing stage, the y coordinate of the ankle joint stays approximately constant with the increase in the number of frames, while the x coordinate of the ankle joint increases with the number of frames that the mobile person moves from left to right. The same valley point can be regarded as the landing of the mobile person’s foot, and the distance and time between the two valleys can be regarded as a progression. The camera frame rate used in this paper is 60 Hz–120 Hz, and each frame is very unblocked. Because the change between image frames is relatively small, the y-direction coordinate is not used for judgment. For each x coordinate, calculate the backward finite difference between the *x*-axis coordinate of the ankle joint, and the previous value is calculated as follows:

(22) [x](t)=x(t)−x(t−1)

where t is used to describe the number of frames, and x(t) is used to represent the position information of the key points on the *x*-axis at the time of frame number *t*. After calculating the position information of each frame, the values are classified according to the International Snow Federation rules.

The three technical actions are mainly judged by the pole position and height of the ski pole and joint angle data. The cycle of sliding movement mainly depends on the combination of the contact and separation of the ankle joint and standing position and the position of the snow stick. For example, in the alternate sliding, the technical action is similar to the walking posture. When the right foot makes contact with the ground to exert force, the right ski stick is at the back position; at this time, the left foot is lifted backward. On the contrary, when the right ski stick and the left foot exert force relative to the front of the body, the left ski stick and the right foot wave backward. When using the double-pole push-and-hold technique, the ski poles on both sides are completely synchronized, and the ankle joints on both sides are also completely synchronized. During the descent, both feet are on the ground at the same time, and there is no stick support for a long time. Therefore, the left and right ankle joint data and the left and right ski pole vertex data are selected as the feature information.

The jitter in the motion curve of key points produces redundant poles, which affect the result to some extent. To overcome this problem, by observation and estimation, a complete motion phase is generally not less than 150 frames. Therefore, any two adjacent troughs that are fewer than 60 frames apart are combined. The same merging method is applied to the ascending and descending phases, although the *y*-axis parameters have changed during the ascending and descending phases. The step size in pixels is defined as the absolute difference between two adjacent high x-value troughs and the Euclidean distance between two troughs. The duration of each step is divided by the frame rate (i.e., 60 FPS–120 FPS) by calculating the number of frames between the two troughs in the rise and fall phases. Finally, the incorrect step size is removed from each video, including the incomplete step size at the beginning and end of the video. By comparing the parameters, the step size that is less than 50 pixels from the median step size of the video is removed.

There are certain differences between different movements due to the changes in the coordinate positions and the angles of the key points of their movement modes, but the trend of athletic movement has rules to follow. Set the position information of feature points as $\left\{x_{1},x_{2},\ldots ,x_{n}\right\}$, and use fast Fourier transform (FFT) to obtain the estimated power spectral density, where the parameters are frequency f and spectrum P(f) [49]. The generated features are shown in Table 2. Each variable has 10 time domain features and 3 frequency domain features, respectively. High-dimensional features will provide rich information, which is conducive to better distinguishing action features and realizing action recognition.

Normalization can prevent one or more dimensions from having a significant impact on the data, allowing each feature to contribute equally to the results, which would play an unbalanced role in training. Common normalization methods include deviation standardization and center standardization. Among them, deviation standardization is a linear transformation of the original data to make the results fall in the range of 0–1. Center standardization is to transform the values of all the features to be converted into a normal distribution with a mean value of 0 and a standard deviation of 1. Compared with central standardization, deviation standardization cannot solve the problem of outliers very well. Therefore, select center standardization to process the generated features.

In human motion recognition, due to the limited number of samples and the features not being prominent, the classification recognition algorithm has high requirements. At present, the human motion recognition algorithm still needs to rely on a large amount of label data when it is applied. The large-scale labeling work not only consumes a lot of human, financial, and material resources but also the data quality is difficult to guarantee, which reduces the training value of the human motion recognition algorithm in ice and snow sports. For the obtained key-point motion curve and angle change curve, this paper uses a support vector machine (SVM) algorithm as collaborative recognition after feature extraction [50]. The formulas of the SVM methods are shown in Formula (7).

(23) $$f\left(x\right)=sign\left(w^{*T}\times k\left(x\right)+b^{*}\right)$$

where $w^{*T}$ is described as the normal vector of the optimal separation hyperplane, and the offset is described as $b^{*}$. SVMs are used as classifiers in collaborative training. The classification confidence of the algorithms is a floating-point number between 0 and 1.

## 4. Experiment and Results

In this part, the effectiveness and robustness of the algorithm are verified by computer simulation and field tests. We conducted posture estimation and motion recognition on the self-made indoor and outdoor cross-country skiing posture simulation video sequence dataset with IMUs and image data to verify the effectiveness of the algorithm. At the same time, in order to verify the effect of this method in pose estimation, we carried out verification on the ski-2d dataset.

### 4.1. Dataset

**Figure 6:**
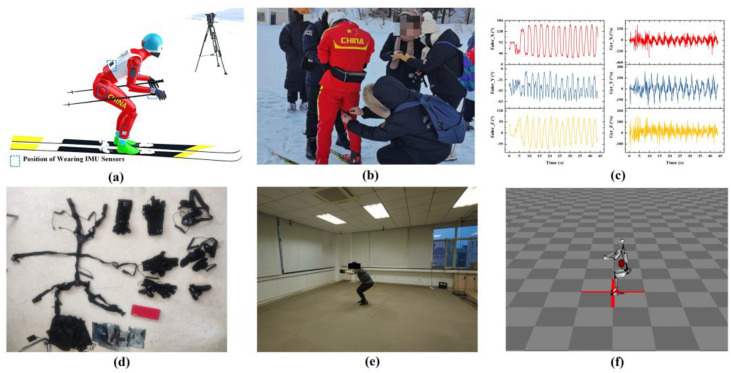
The cross-country skiing dataset and sample image display. (**a**) shows the outdoor dataset collection method. (**b**) shows the sensor worn by the experimental personnel. (**c**) shows outdoor sensor data. (**d**) shows the indoor dataset acquisition mode. (**e**) shows the indoor dataset acquisition process. (**f**) shows the result of the indoor dataset.

The ski-2d dataset mainly collects semi-professional skier videos recorded from different angles in different weather conditions [25]. It is suitable for computer vision and sports science to conduct in-depth research on skiing. At the same time, in order to improve the practical application value for cross-country skiing, we collected and calibrated cross-country skiing videos recorded from multiple angles in laboratory and competition environments for verification experiments as a self-made dataset in Figure 6. The outdoor video sequence dataset comes from the Chinese National Biathlon Team and was recorded at the Bayi Ski Resort in Mudanjiang City, Heilongjiang Province. The athletes wore IMU sensors and collected multi-angle videos, as shown in Figure 6a–c. The video frame rate is 60 Hz, collected from six athletes of different heights and ages. We consider the IMU data to be the true value of the dataset and the test conditions are highly consistent with the actual competition situation. The indoor dataset uses the Noitom dynamic capture system to collect the athlete’s postural information and video recording in Figure 6d–f [51]. We imitated the actions of standing skiing and sitting skiing, recorded the video with a monocular camera, and annotated the sensor data as the true value of the indoor video at a 60 Hz frame rate.

When inputting athlete videos for action recognition, the algorithm in this paper cannot distinguish between different athletes, and all athletes are marked as a person category. Therefore, if more than one person is recognized at the same time, there may be confusion between the results. Therefore, in the actual test, we try to ensure that only one athlete appears in the image during the capture. If the video contains multiple targets, we trim the video to reduce the effect of multiple targets. In terms of video resolution, the method in this paper has universal adaptability, and 360p (640 × 360) to 4k (4096 × 2160) resolution videos have been tested. At these resolutions, the proposed method can accurately implement attitude estimation and motion detection. In terms of frame rate, according to reference [18], we recommend that the video frame rate should not be less than 50 Hz. The algorithm in this paper has no requirements as to the input video file format. The test results show that different video formats have little effect on attitude estimation and motion detection. Considering the generalization ability, this article uses part of the COCO dataset in the training of the two-dimensional posture recognition module. However, if the clothing and background colors of the athletes are similar, it is still possible to fail to identify the athlete category, resulting in the inability to achieve posture estimation and motion recognition.

### 4.2. Metrics

For the evaluation method, we mainly evaluate the results of the pose estimation by the percentage of correct key points (PCK) [52], the mean joint position error (MPJPE) [53], and the mean absolute error (MAE).

The PKC is used to verify the success rate of different methods for the same image or video recognition by calculating the number of joint points detected and the corresponding number of ground true points among a given number of images or targets. The formula is as follows:

(24) $$PCK=\frac{Num_{det}}{Num_{gt}}$$

where $Num_{det}$ and $Num_{gt}$ are used to represent the total number of keys detected and the total number of keys in the target.

In the validation experiment, for the results of 2D and 3D joint point estimation, we use the percentage of successfully predicted key points and the mean joint position error (MPJPE) of each joint position to evaluate the detection effect of different algorithms [30]. For each frame *f* and target *x*, MPJPE is calculated as follows:

(25) $$MPJPE\left(f,\;x\right)=\frac{1}{S}\sum_{i=1}^{s}m_{det}^{f}\left(x_{i}\right)-m_{gt}^{f}{\left(x_{i}\right)}_{2}$$

where all joint points of the dataset are represented by S. $m_{det}^{f}$ and the $m_{gt}^{f}$ are used to represent the estimation results of the algorithm and ground truth in each frame, respectively. From the formula, the results of 2D and 3D pose estimation of the algorithm consist of the ground truth when the MPJPE is small.

At the same time, for example, the athlete’s angle information is very significant in guiding training. Therefore, we introduce the mean absolute error (MAE) widely used in ski competition analysis to compare the angle information calculated by different algorithms, which is called the mean absolute error of angle (MAEA). The MAEA calculation is as follows

(26) $$MAE=\frac{1}{m}\sum \begin{array}{c} m\\ i=1 \end{array}\left|x_{gt}^{i}-x_{det}^{i}\right|$$

where the number of all targets is expressed by m. $x_{det}^{i}$ and $x_{gt}^{i}$ are used to represent the estimated angle result detected by our method and the ground truth in each frame.

### 4.3. Experiment Result of Pose Estimation

In the simulation, the computer hardware environment is GeForce 1060, and the default environment is PyTorch. Table 3 shows the number of key points successfully detected and the (PCK) of the three methods. Table 4 shows the average joint error of our algorithm and other mainstream human posture estimation methods including OpenPose [14] and SimDr [42] on the ski-2d dataset. OpenPose is a heatmap-based method, while SimDR is based on regression. By comparing these two algorithms, it can be verified that our algorithm has advantages in pose estimation compared to the method based on heatmap and the method based on regression.

From Table 3 and Table 4, we can see that our algorithm has a success rate of 95% and it successfully identifies almost all the key points. At the same time, our algorithm has a small average joint error in Table 4. Compared to the human posture estimation algorithms, our algorithm has a smaller error under the condition that almost all key points are successfully identified. Therefore, our method has better performance compared to other commonly used pose estimation algorithms. The pose estimation result of our algorithm still has errors. We speculate that the reason for this is related to the view of our dataset. There are many scenes in the dataset where cameras and cross-country trails are kept horizontal. When cameras and moving athletes are kept horizontal, the occlusion situation is relatively severe, and the recognition error of key points on the occluded side is relatively large, which also indicates that self-occlusion is a major challenge in pose estimation. Although there are errors in key-point estimation, the overall motion trend of the key points remains accurate, providing valuable feature information for motion recognition.

**Figure 7:**
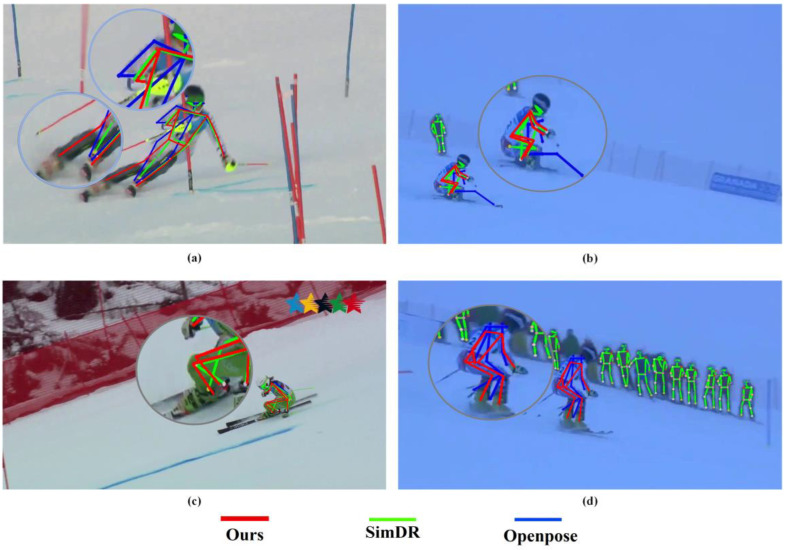
The results of different algorithms in the same video sequence. In (**a**), the algorithm in this article has more accurate results for the right foot and elbow. In (**b**), the position of the method in this paper is relatively accurate, and the OpenPose estimation shows key-point drift. In (**c**), the OpenPose method was not recognized and the left and right leg points of the SimDR method were confused. In (**d**), our method achieves the accuracy of gesture recognition, while OpenPose cannot accurately identify some key points, and SimDR cannot identify the target.

In the video sequence shown in Figure 7, different methods produce different recognition results for the same image. The red curve represents the method in this paper, the green curve represents the SimDR method, and the blue curve represents the results of the OpenPose algorithm. In Figure 7a, our algorithm has more accurate results on the right foot and elbow. In Figure 7b, the posture of our method is relatively accurate, and OpenPose experienced key-point drift. In Figure 7c, the OpenPose method failed to recognize the key points, and the left and right leg points were confused by the SimDR method. In Figure 7d, our method implemented pose recognition accurately, while some key points were not recognized accurately by OpenPose, and SimDR failed to recognize the target. Therefore, our algorithm has the best recognition results, such as knee, ankle, and elbow joint recognition, which are better than the comparison algorithms.

**Figure 8:**
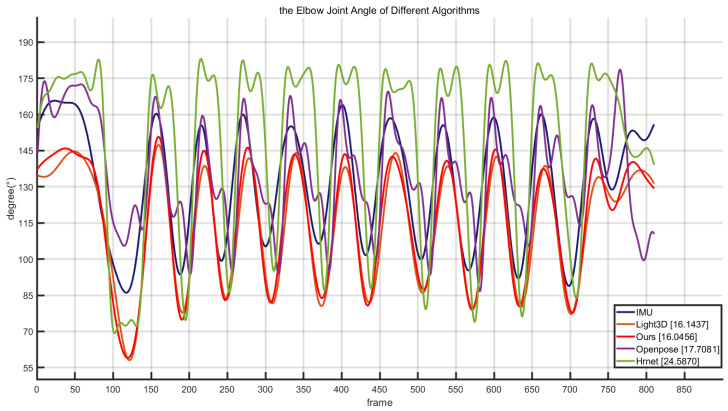
Elbow angle results estimated by different algorithms using the same video sequence.

In cross-country skiing, the joint angle information obtained from the joint point coordinates provides technical support for the scientific training of athletes [54,55]. At the same time, the joint angle information is also an important feature for subsequent action recognition. To verify the application value of the algorithm in cross-country skiing, we evaluated the results of four methods for estimating the athlete’s elbow angle. From the joint angle curves identified by different methods in Figure 8, we can see that the joint angle calculated by our method is more similar to the angle information collected by the sensor, and the change process of the curve is basically consistent with the sensor data. The joint angle calculation effect of light3d is second to our method and has a good effect. Both of these algorithms obtain 3D depth information through 2D estimation, so obtaining more accurate 2D coordinate point information is conducive to improving the accuracy of depth information estimation. The other two methods, OpenPose and HRNet [56], due to the lack of depth information, can only accurately estimate the joint angle or angle change trend when the target and camera positions are kept at a basic level. At the same time, compared with the four curves, our results and the IMU results have the most similar trend. Therefore, our method can obtain features that are more similar to the true value when dividing the features, which is conducive to the realization of cross-country skiing movement recognition.

Table 5 describes the joint MAE of the different algorithms in the test image sequence. The joint MAE of this work is the smallest, the joint error of light3d is the second smallest, the joint error of OpenPose is higher, and the MAE of HRNet is the largest. Therefore, compared to other algorithms, our method has the best accuracy in estimating the joint angle of monocular ice and snow motion.

Through the verification experiments using multiple algorithms on the ski dataset and the self-made cross-country skiing dataset in this paper, the algorithm in this paper has a high recognition success rate (95%) and a low joint accuracy error (0.0894) in two-dimensional joint points. In the simulation of 3D joint angle estimation, the joint error of this method is 0.1° lower than that of the comparison algorithm light3d, which has the best joint accuracy. Therefore, the joint detection and estimation algorithm proposed in this paper has application value in cross-country skiing posture detection and scientific training.

### 4.4. Simulation Results of Motion Recognition

**Figure 9:**
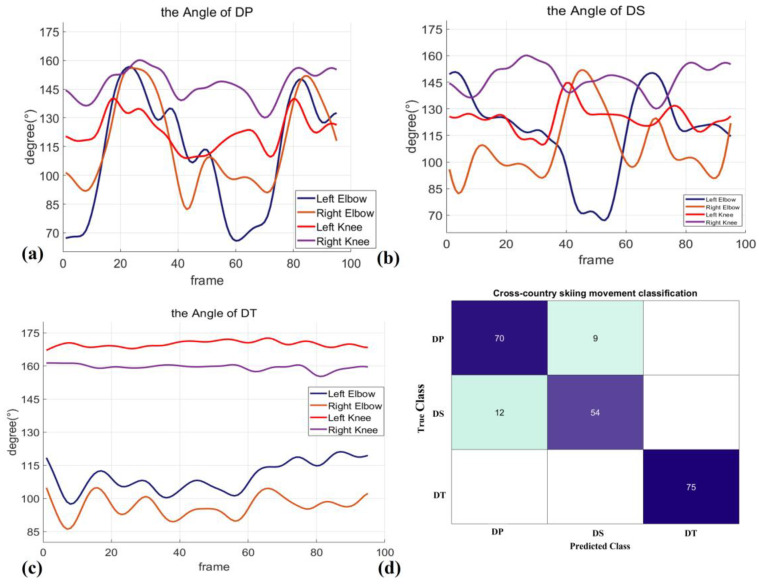
The joint characteristics and motion recognition results of different sub-actions. (**a**) shows the trend of change in elbow angle and knee stick in the DP action. (**b**) shows the change trend of elbow angle and knee stick in the DS action. (**c**) shows the change trend of the elbow angle and knee stick in the DT action. (**d**) shows the motion recognition results of different sub-actions.

In cross-country skiing motion recognition, due to the limited number of samples, the high dimension of the samples, and the rich and irregular posture of the athletes, there is a high demand for the accuracy and robustness of the classification recognition algorithm. The feature vectors and their motion types are obtained by calculating the key-point position data and joint angle data identified by the algorithm in this paper, including 79 groups of DP motion data, 66 groups of DS motion data, and 75 groups of DT data. Through cross-validation, the training data set is divided into five batches, and the accuracy of each batch is estimated to prevent over-fitting. Each time, four batches are randomly selected as a training set and the rest as a test set. The result is shown in the figure, and the recognition accuracy is 90.5%. Table 5 describes the accuracy rate of action recognition in this paper and other methods based on wearable sensors. From Table 6, it can be seen that the use of multiple IMU sensors to achieve action classification is the best. The recognition result of our method based on optical sensors is better than that of the method based on wearable gyroscope sensors. The method that relies solely on image recognition and does not extract features has the lowest recognition success rate, with a quarter failing to make an accurate identification. When using SVM to implement action recognition, the five disjoints are used in cross-validation and reserve 10% of the data as the validation set. The confusion matrix is shown in Figure 9d.

In general, the motion recognition accuracy of the method used in this paper is similar to that of the methods based on wearable sensors [57,58,59]. Therefore, our method provides a reference method for image-based cross-country skiing motion recognition.

### 4.5. Ablation Experiment

In this part, we performed ablation experiments to test the effect of improving candidate anchors for two-dimensional estimation and improving two-dimensional poses for three-dimensional estimation.

For the selection of candidate regions, by modifying the anchor size, our method improves the success rate and the mean average precision (mAP) of the candidate region for athletes [60]. Table 7 shows the results of our method compared to the results of the original YOLO and other recognition algorithms. Compared to the original YOLO-v5, the mAP of our method is improved by 0.02, and the AP of our algorithm is better than Shuffle Net. Therefore, it is effective in improving the detection of candidate regions.

Using the COCO dataset, our method is compared with the heatmap-based estimation method, and the results are shown in Table 8. OpenPose and HRNet use a heatmap, while SimDR and our method are based on regression. From the data in the table, it can be seen that the heatmap-based methods are limited by the problem of heatmap-based estimation bias, and their accuracy is lower than that of our method and SimDR. Both our method and SimDR have good detection accuracy. The AP of OpenPose is 0.618 and the accuracy of our method is 0.716. Therefore, compared to the heatmap-based method, the regression-based method has a higher accuracy in key-point estimation.

In order to verify the effect of the two-dimensional input on the three-dimensional recognition result, our method is compared with the three-dimensional estimation methods based on a heatmap. Using the same three-dimensional estimation network, the effect of different two-dimensional key-point inputs on the three-dimensional pose estimation is described in Table 9. For the same video sequence, in the same three-dimensional estimation network, different two-dimensional pose information can affect the three-dimensional estimation results. The more accurate the two-dimensional results, the better the three-dimensional pose estimation effect.

Therefore, based on the experimental results, improved candidate anchors for two-dimensional pose estimation, two-dimensional pose estimation based on regression, and more accurate two-dimensional key-point input can improve the pose estimation effect.

## 5. Discussion

The purpose of this study is to verify the feasibility of the motion detection and analysis system for cross-country skiing technology based on a visible light sensor. Our priority is the ease of use of the system and the accuracy of the motion recognition. For ease of use, we have developed a motion detection method based on a monocular camera, which does not need to calibrate the environment in advance. This method does not interfere with the athletes’ wearable sensors and complex environmental calibration and has good usability. To improve the accuracy of cross-country skiing motion recognition, we adopted the bottom-up key-point detection method, which detects the athlete’s target and key points simultaneously, the three-dimensional posture estimation method, and the feature extraction and classification method based on cross-country skiing characteristics to implement the motion recognition.

The heatmap-based recognition method has insufficient accuracy in outdoor environments and is not suitable for estimating postures in outdoor conditions such as those of cross-country skiing. To solve these problems, we adopted the key-point detection method, which simultaneously detects human posture and key points, and uses four feature layers of different output grid scales to directly identify key points and targets. By comparison with other algorithms, as shown in Table 2 and Table 3, it has better identification accuracy on a public dataset and a self-made dataset. Through ablation experiments, it can be demonstrated that improving the candidate anchor and pose estimation using the regression method can improve the accuracy of two-dimensional pose estimation.

The two-dimensional key points lack depth information, and the positioning of key points is not accurate enough, which easily leads to errors in the calculation of key-point position and joint angle. In order to improve the accuracy, we built a depth information estimation network and used the position deviation loss and rotation compensation loss of key points as the loss function to implement the three-dimensional estimation of key points. It can be seen from Figure 3 and Table 4 that the method in this paper has higher accuracy compared to the two-dimensional estimation method and other three-dimensional key-point estimation methods. It also shows that more accurate 2D key-point information can improve the accuracy of 3D estimation.

Finally, the typical characteristics of cross-country skiing are determined by analyzing the action stages and the sub-actions of cross-country skiing. In cross-country skiing, the elbows and knees move differently in different sub-actions. By extracting features from the key-point location and angle information obtained from the image sensor, the characteristics of the sub-actions have a greater difference, and the motion detection effect is similar to that of the sensor-based method. As shown in Table 6, when the joint motion data are fed directly into the classifier, the recognition accuracy is 20% lower than that of our method. All these improvements have been tested in the snow field. Therefore, the optical sensor-based non-contact motion recognition method designed in this paper has application value in the scientific training of cross-country skiing.

## 6. Conclusions

In this paper, a cross-country skiing kinematics information acquisition system based on optical sensors was designed to collect and analyze the movements of cross-country skiing athletes. The system is able to collect information on the athlete’s path, posture, and stage of movement in a non-contact manner. In order to solve the problems such as the difficulty of extracting objects in the outdoor field, the occlusion of the view angle, and the change in the motion attitude in the attitude estimation, we proposed a 3D attitude estimation method by the cooperative recognition of key points and objects. By simultaneously recognizing the target and key points in four feature output grid scales, more accurate key-point recognition results can be obtained compared with the heatmap method. The accurate depth information of key points is obtained by position deviation loss and rotation compensation loss, and the accurate recognition and classification of action stages and sub-actions are achieved by cross-country skiing characteristics and feature extraction. The experimental results show that the algorithm is more effective than the other three two-dimensional position estimation methods in the ski-2d dataset and the four three-dimensional position estimation methods in our dataset. It also has a smaller joint position error and more correct key points than the benchmark two-dimensional estimation algorithm. In 3D estimation, the average joint error is less than the benchmark method (0.1°). The correct rate of sub-action classification reaches 90%, which is similar to the motion recognition method based on wearable sensors. Therefore, the algorithm has application value in the scientific training of cross-country skiers and has been applied to the training of professional ski teams.

In the future, we will improve the 3D posture estimation network method to improve the detection accuracy of 3D posture estimation. At the same time, future work will focus on improving the accuracy of motion detection, improving the real-time performance of the algorithm, and providing professional training suggestions using the acquired data.

## Figures and Tables

**Table 1:** The characteristics of the different cross-country skiing sub-actions.

Category	Features
Double Poling (DP)	Two sets of elbow joints and two sets of knee joints in the same way.
Diagonal Stride (DS)	The movements of the two elbow joints and the two knee joints are opposite.
Downhill Techniques (DT)	The elbow and knee joints are the same, and the angle does not change much over time.

**Table 2:** The characteristics of sub-actions.

Characteristic Quantity	Definition
Maximum	$Max=\max\left\{x_{1},x_{2},\ldots ,x_{n}\right\}$
Minimum	$Min=\min\left\{x_{1},x_{2},\ldots ,x_{n}\right\}$
Average	$mean=\frac{1}{n}\overset{n}{\underset{i=1}{\sum }}X_{i}$
Peak	pk=Max−Min
Standard deviation	$st=\sqrt{\frac{1}{n}\overset{n}{\underset{i=1}{\sum }}{\left(X_{i}-mean\right)}^{2}}$
Peakedness	$ku=\frac{\sum_{i=1}^{n}{\left(X_{i}-mean\right)}^{4}}{\left(n-1\right)\times st^{4}}$
Skewness	$sk=\frac{\sum_{i=1}^{n}{\left(X_{i}-mean\right)}^{3}}{n\times st^{3}}$
Root mean square	$rm=\sqrt{\frac{1}{n}\overset{n}{\underset{i=1}{\sum }}{\left|X_{i}\right|}^{2}}$
Root mean square factor	$S=rm/\frac{1}{n}\overset{n}{\underset{i=1}{\sum }}\left|X_{i}\right|$
Peak factor	C=pk/rm
Center of gravity frequency	$FC=\frac{\int_{0}^{+\infty }fP\left(f\right)df}{\int_{0}^{+\infty }P\left(f\right)df}$
Mean square frequency	$MSF=\frac{\int_{0}^{+\infty }f^{2}P\left(f\right)df}{\int_{0}^{+\infty }P\left(f\right)df}$
Frequency variance	$VF=\frac{\int_{0}^{+\infty }{\left(f-FC\right)}^{2}P\left(f\right)df}{\int_{0}^{+\infty }P\left(f\right)df}$

**Table 3:** The success detected number and PCK of different algorithms.

Algorithm	OpenPose	SimDr	Ours
**Key-Point Numbers**	714	714	714
**Success Detected**	547	714	684
**PCK**	76.61%	100%	95.31%

**Table 4:** The mean per joint position error of different algorithms.

Algorithm	OpenPose	SimDr	Ours
**MPJPE**	0.081	0.098	0.089

**Table 5:** The MAE of the elbow angle of different algorithms in the test image sequence.

Algorithm	OpenPose	HRNet	Light	Ours
**MAE (°)**	17.708	24.587	16.144	16.046

**Table 6:** Motion recognition accuracy of different methods.

Method	Wearable Gyroscope Sensors [57]	Three IMU Sensors [58]	Four IMU Sensors [59]	Ours without Feature Extraction	Ours
**Accuracy**	86.82%	95.4%	98%	76.8%	90.5%

**Table 7:** The mAP in different algorithms.

Algorithm	Shuffle Net	YOLO	Ours
**mAP**	0.605	0.692	0.711

**Table 8:** The AP in different algorithms.

Algorithm	OpenPose	HRNet	SimDR	Ours
**AP**	0.618	0.705	0.708	0.716

**Table 9:** The MAE in different algorithms.

Algorithm	Light3D	Ours
**MAE (°)**	16.144	16.046

## Data Availability

The data presented in this study are available on request from the corresponding author.

## References

[1] Stöggl T., Müller E., Lindinger S. (2008). Biomechanical Comparison of the Double-Push Technique and the Conventional Skate Skiing Technique in Cross-Country Sprint Skiing. J. Sport. Sci..

[2] Cust E.E., Sweeting A.J., Ball K., Robertson S. (2018). Machine and Deep Learning for Sport-Specific Movement Recognition: A Systematic Review of Model Development and Performance. J. Sport. Sci..

[3] Ruiz-García I., Navarro-Marchal I., Ocaña-Wilhelmi J., Palma A.J., Gómez-López P.J., Carvajal M.A. (2021). Development and Evaluation of a Low-Drift Inertial Sensor-Based System for Analysis of Alpine Skiing Performance. Sensors.

[4] Yang Y., Hou X., Geng W., Mu J., Zhang L., Wang X., He J., Xiong J., Chou X. (2022). Human Movement Monitoring and Behavior Recognition for Intelligent Sports Using Customizable and Flexible Triboelectric Nanogenerator. Sci. China Technol. Sci..

[5] Fasel B., Spörri J., Gilgien M., Boffi G., Chardonnens J., Müller E., Aminian K. (2016). Three-Dimensional Body and Centre of Mass Kinematics in Alpine Ski Racing Using Differential GNSS and Inertial Sensors. Remote Sens..

[6] Yu G., Jang Y., Kim J., Kim J., Kim H., Kim K., Panday S. (2016). Potential of IMU Sensors in Performance Analysis of Professional Alpine Skiers. Sensors.

[7] Kulikajevas A., Maskeliunas R., Damaševičius R. (2021). Detection of Sitting Posture Using Hierarchical Image Composition and Deep Learning. PeerJ Comput. Sci..

[8] Skach S., Stewart R., Healey P.G.T. Smart Arse: Posture Classification with Textile Sensors in Trousers. Proceedings of the 20th ACM International Conference on Multimodal Interaction.

[9] Gupta R., Gupta S.H., Agarwal A., Choudhary P., Bansal N., Sen S. A Wearable Multisensor Posture Detection System. Proceedings of the 2020 4th International Conference on Intelligent Computing and Control Systems (ICICCS).

[10] Gupta R., Gupta A., Aswal R. Detection of Poor Posture Using Wearable Sensors and Unsupervised Learning. Proceedings of the 2021 7th International Conference on Advanced Computing and Communication Systems (ICACCS).

[11] Ryselis K., Blažauskas T., Damaševičius R., Maskeliūnas R. (2022). Computer-Aided Depth Video Stream Masking Framework for Human Body Segmentation in Depth Sensor Images. Sensors.

[12] Rangasamy K., As’ari M.A., Rahmad N.A., Ghazali N.F., Ismail S. (2020). Deep Learning in Sport Video Analysis: A Review. TELKOMNIKA Telecommun. Comput. Electron. Control..

[13] International Ski Federation (Fédération Internationale de Ski—FIS) (2023). In International Year Book and Statesmen’s Who’s Who.

[14] Cao Z., Simon T., Wei S.-E., Sheikh Y. Realtime Multi-Person 2D Pose Estimation Using Part Affinity Fields. Proceedings of the 2017 IEEE Conference on Computer Vision and Pattern Recognition (CVPR).

[15] Bittner M., Yang W.-T., Zhang X., Seth A., van Gemert J., van der Helm F.C.T. (2022). Towards Single Camera Human 3D-Kinematics. Sensors.

[16] Nguyen H.-C., Nguyen T.-H., Scherer R., Le V.-H. (2022). Unified End-to-End YOLOv5-HR-TCM Framework for Automatic 2D/3D Human Pose Estimation for Real-Time Applications. Sensors.

[17] Hu K., Ding Y., Jin J., Xia M., Huang H. (2022). Multiple Attention Mechanism Graph Convolution HAR Model Based on Coordination Theory. Sensors.

[18] Pellegrini B., Sandbakk Ø., Stöggl T., Supej M., Ørtenblad N., Schürer A., Steiner T., Lunina A., Manhard C., Liu H. (2021). Methodological Guidelines Designed to Improve the Quality of Research on Cross-Country Skiing. J. Sci. Sport Exerc..

[19] Cenedese A., Susto G.A., Terzi M. A Parsimonious Approach for Activity Recognition with Wearable Devices: An Application to Cross-Country Skiing. Proceedings of the 2016 European Control Conference (ECC).

[20] Rindal O., Seeberg T., Tjønnås J., Haugnes P., Sandbakk Ø. (2017). Automatic Classification of Sub-Techniques in Classical Cross-Country Skiing Using a Machine Learning Algorithm on Micro-Sensor Data. Sensors.

[21] Marsland F., Mackintosh C., Holmberg H.-C., Anson J., Waddington G., Lyons K., Chapman D. (2017). Full Course Macro-Kinematic Analysis of a 10 Km Classical Cross-Country Skiing Competition. PLoS ONE.

[22] Johansson M., Korneliusson M., Lawrence N.L. (2019). Identifying Cross Country Skiing Techniques Using Power Meters in Ski Poles. Communications in Computer and Information Science.

[23] Meyer F., Prenleloup A., Schorderet A. (2019). Development of a New Embedded Dynamometer for the Measurement of Forces and Torques at the Ski-Binding Interface. Sensors.

[24] Krüger A., McAlpine P., Borrani F., Edelmann-Nusser J. (2011). Determination of Three-Dimensional Joint Loading within the Lower Extremities in Snowboarding. Proc. Inst. Mech. Eng. Part H J. Eng. Med..

[25] Bachmann R., Sporri J., Fua P., Rhodin H. Motion Capture from Pan-Tilt Cameras with Unknown Orientation. Proceedings of the 2019 International Conference on 3D Vision (3DV).

[26] Gastaldi L., Mauro S., Pastorelli S. (2016). Analysis of the Pushing Phase in Paralympic Cross-Country Sit-Skiers—Class LW10. J. Adv. Res..

[27] Gløersen Ø N. (2014). Quantitative Technique Analysis in XC-Skiing. Master’s Thesis.

[28] Li W., Liu H., Tang H., Wang P., Van Gool L. MHFormer: Multi-Hypothesis Transformer for 3D Human Pose Estimation. Proceedings of the 2022 IEEE/CVF Conference on Computer Vision and Pattern Recognition (CVPR).

[29] Yamamoto K., Nishino T., Bale R., Shimada T., Miyamoto N., Tsubokura M. (2022). Numerical Study of Transient Aerodynamic Forces Acting on a Ski Jumper Considering Dynamic Posture Change from Takeoff to Landing. Sport. Biomech..

[30] Wang P., Li Z., Hou Y., Li W. Action Recognition Based on Joint Trajectory Maps Using Convolutional Neural Networks. Proceedings of the 24th ACM international conference on Multimedia.

[31] Li Y., Xia R., Liu X. (2020). Learning Shape and Motion Representations for View Invariant Skeleton-Based Action Recognition. Pattern Recognit..

[32] Tran D., Bourdev L., Fergus R., Torresani L., Paluri M. Learning Spatiotemporal Features with 3D Convolutional Networks. Proceedings of the 2015 IEEE International Conference on Computer Vision (ICCV).

[33] Yang W., Ouyang W., Li H., Wang X. End-to-End Learning of Deformable Mixture of Parts and Deep Convolutional Neural Networks for Human Pose Estimation. Proceedings of the 2016 IEEE Conference on Computer Vision and Pattern Recognition (CVPR).

[34] Bo L., Yuchao D., Xuelian C., Huahui C., Yi L., Mingyi H. Skeleton Based Action Recognition Using Translation-Scale Invariant Image Mapping and Multi-Scale Deep CNN. Proceedings of the 2017 IEEE International Conference on Multimedia & Expo Workshops (ICMEW).

[35] Wang J., Huang S., Wang X., Tao D. Not All Parts Are Created Equal: 3D Pose Estimation by Modeling Bi-Directional Dependencies of Body Parts. Proceedings of the 2019 IEEE/CVF International Conference on Computer Vision (ICCV).

[36] Ma X., Su J., Wang C., Ci H., Wang Y. Context Modeling in 3D Human Pose Estimation: A Unified Perspective. Proceedings of the 2021 IEEE/CVF Conference on Computer Vision and Pattern Recognition (CVPR).

[37] Pellegrini B., Zoppirolli C., Bortolan L., Zamparo P., Schena F. (2014). Gait Models and Mechanical Energy in Three Cross-Country Skiing Techniques. J. Exp. Biol..

[38] Losnegard T. (2019). Energy System Contribution during Competitive Cross-Country Skiing. Eur. J. Appl. Physiol..

[39] Norman R.W., Komi P.V. (1987). Mechanical Energetics of World Class Cross-Country Skiing. Int. J. Sport Biomech..

[40] Wang C.-Y., Bochkovskiy A., Liao H.-Y.M. Scaled-YOLOv4: Scaling Cross Stage Partial Network. Proceedings of the 2021 IEEE/CVF Conference on Computer Vision and Pattern Recognition (CVPR).

[41] Wang C., Zhang F., Zhu X., Ge S.S. (2022). Low-Resolution Human Pose Estimation. Pattern Recognit..

[42] McNally W., Vats K., Wong A., McPhee J. (2022). Rethinking Keypoint Representations: Modeling Keypoints and Poses as Objects for Multi-Person Human Pose Estimation. Lecture Notes in Computer Science.

[43] Li Y., Yang S., Liu P., Zhang S., Wang Y., Wang Z., Yang W., Xia S.-T. (2022). SimCC: A Simple Coordinate Classification Perspective for Human Pose Estimation. Lecture Notes in Computer Science.

[44] Osokin D. Real-Time 2D Multi-Person Pose Estimation on CPU: Lightweight OpenPose. Proceedings of the 8th International Conference on Pattern Recognition Applications and Methods.

[45] Mehta D., Sotnychenko O., Mueller F., Xu W., Sridhar S., Pons-Moll G., Theobalt C. Single-Shot Multi-Person 3D Pose Estimation from Monocular RGB. Proceedings of the 2018 International Conference on 3D Vision (3DV).

[46] He K., Zhang X., Ren S., Sun J. Deep Residual Learning for Image Recognition. Proceedings of the 2016 IEEE Conference on Computer Vision and Pattern Recognition (CVPR).

[47] Mehta D., Rhodin H., Casas D., Fua P., Sotnychenko O., Xu W., Theobalt C. Monocular 3D Human Pose Estimation in the Wild Using Improved CNN Supervision. Proceedings of the 2017 International Conference on 3D Vision (3DV).

[48] Martinez J., Hossain R., Romero J., Little J.J. A Simple Yet Effective Baseline for 3d Human Pose Estimation. Proceedings of the 2017 IEEE International Conference on Computer Vision (ICCV).

[49] Dargie W. Analysis of Time and Frequency Domain Features of Accelerometer Measurements. Proceedings of the 18th International Conference on Computer Communications and Networks.

[50] Schuldt C., Laptev I., Caputo B. Recognizing Human Actions: A Local SVM Approach. Proceedings of the 17th International Conference on Pattern Recognition.

[51] Shuai Z., Dong A., Liu H., Cui Y. (2022). Reliability and Validity of an Inertial Measurement System to Quantify Lower Extremity Joint Angle in Functional Movements. Sensors.

[52] Newell A., Yang K., Deng J. (2016). Stacked Hourglass Networks for Human Pose Estimation. Computer Vision—ECCV 2016.

[53] Ionescu C., Papava D., Olaru V., Sminchisescu C. (2014). Human3.6M: Large Scale Datasets and Predictive Methods for 3D Human Sensing in Natural Environments. IEEE Trans. Pattern Anal. Mach. Intell..

[54] Bere T., Bahr R. (2014). Injury Prevention Advances in Alpine Ski Racing: Harnessing Collaboration with the International Ski Federation (FIS), Long-Term Surveillance and Digital Technology to Benefit Athletes. Br. J. Sport. Med..

[55] Bucher Sandbakk S., Supej M., Sandbakk Ø., Holmberg H.-C. (2013). Downhill Turn Techniques and Associated Physical Characteristics in Cross-Country Skiers. Scand. J. Med. Amp; Sci. Sport..

[56] Sun K., Xiao B., Liu D., Wang J. Deep High-Resolution Representation Learning for Human Pose Estimation. Proceedings of the 2019 IEEE/CVF Conference on Computer Vision and Pattern Recognition (CVPR).

[57] Jang J., Ankit A., Kim J., Jang Y.J., Kim H.Y., Kim J.H., Xiong S. (2018). A Unified Deep-Learning Model for Classifying the Cross-Country Skiing Techniques Using Wearable Gyroscope Sensors. Sensors.

[58] Zhang Z., Yang Y., Chen L., He J. (2022). Cross-country skiing technique action recognition based on inertial sensing. Transducer Microsyst. Technol..

[59] Tjønnås J., Seeberg T.M., Rindal O.M.H., Haugnes P., Sandbakk Ø. (2019). Assessment of Basic Motions and Technique Identification in Classical Cross-Country Skiing. Front. Psychol..

[60] Lin T.-Y., Maire M., Belongie S., Hays J., Perona P., Ramanan D., Dollár P., Zitnick C.L. (2014). Microsoft COCO: Common Objects in Context. Computer Vision—ECCV 2014.

